# Balancing the evidence: An update on analgesic use in rheumatic and musculoskeletal diseases

**DOI:** 10.3389/fdsfr.2023.1117674

**Published:** 2023-02-16

**Authors:** Yun-Ting Huang, Craig McCarthy, Meghna Jani

**Affiliations:** ^1^ Centre for Epidemiology Versus Arthritis, Centre for Musculoskeletal Research, University of Manchester, Manchester, United Kingdom; ^2^ NIHR Manchester Biomedical Research Centre, University of Manchester, Manchester, United Kingdom

**Keywords:** rheumatic and musculoskeletal diseases (RMDs), pharmacological treatment, paracetamol (acetaminophen), non-steroidal anti-inflammatory drugs (NSAIDs), opioids, antidepressants, gabapentinoids

## Abstract

Pain management has been a challenging issue for people living with rheumatic and musculoskeletal diseases (RMDs) and health professionals for decades. Pharmacological treatments remain a core element of pain management of inflammatory arthritis and osteoarthritis. Yet balancing the benefits/harms in pain management within RMDs can be difficult to navigate due to limited effective options, and emerging adverse events in a population where individual risk is important to consider due to patient multimorbidity, immunosuppression and polypharmacy. Paracetamol and non-steroidal anti-inflammatory drugs (NSAIDs) analgesics are widely used among RMD patients, however both classes of drugs have been associated with new safety concerns in the last two decades. Perhaps as a result in combination with multifactorial influences, opioid prescribing has increased from the 2000s–2010s in the majority of RMD focussed studies, accompanied with a rising trend of long-term opioid use, despite limited evidence on efficacy. Gabapentinoids have also shown increasing trends more recently, despite an unclear role in chronic pain management for RMDs within current guidelines. Antidepressants are recommended as the first line of pharmacological treatment of chronic primary pain (e.g., fibromyalgia) by the latest National Institute for Health and Care Excellence (NICE) guideline released in April 2021. This concise narrative review will discuss pharmacological options for pain management, based on the latest evidence that includes the main analgesic drug classes: paracetamol, NSAIDs, opioids, antidepressants, and gabapentinoids. We will discuss the efficacy of these analgesics in RMDs and emerging safety concerns to enable more informed shared decisions with patients commencing such medications.

## Introduction

Pain management in people living with RMDs has been a challenge to address for health professionals for decades. From the 2000s–2010s (i.e., 2010-2019), there has been a considerable increase in the prescriptions of antidepressants (Ivanova et al., 2011; John et al., 2016), gabapentinoids (Torrance et al., 2020; Kuehn, 2022), and opioids (Kalso et al., 2004; Jani et al., 2020; Anastasiou and Yazdany, 2022) for pain management, especially chronic pain, worldwide. Recommendations for chronic pain internationally can vary considerably and are heterogeneous, depending on underlying conditions. According to the European Alliance of Associations for Rheumatology (EULAR) recommendations, pharmacological treatments continue to remain important in pain management of inflammatory arthritis and osteoarthritis (OA). The recent NICE guideline from April 2021 places more emphasis on non-pharmacological treatments and recommends antidepressants as the first-line pharmacological treatment of chronic primary pain (e.g., fibromyalgia) (National Institute for Health and Care Excellence, 2021a). The strategy for chronic pain caused by an underlying condition [e.g., rheumatoid arthritis (RA)] however is unclear in this guideline. Balancing the benefits and harms of pain medications within different RMDs can be difficult due to limited effective therapeutic options. These need to be considered with emerging adverse events in a population where individual risk is especially important because of the presence of multimorbidity, immunosuppression and polypharmacy.

Despite the widespread use of paracetamol and NSAIDs for pain control, evidence of new safety concerns has emerged in the last two decades. Other analgesics such as opioids and antidepressants have also drawn many investigations and discussion, while a few research focuses on the use of gabapentinoids. This concise narrative review will discuss pharmacological options for pain management in RMDs based on the latest evidence, with an emphasis on efficacy, potential adverse effects and safety concerns. Five main drug classes are included in this review and introduced in the following order: paracetamol, NSAIDs, opioids, antidepressants and gabapentinoids.

## Paracetamol

Paracetamol is widely recommended for pain management, including by the World Health Organisation (WHO, 2019; Freo et al., 2021). Conditional recommendations of paracetamol are made by different organisations for pain conditions, including axial spondyloarthritis (AxSpA), low back pain (LBP), OA, musculoskeletal pain, headache, and cancer pain (Freo et al., 2021). For example, according to the NICE guideline, paracetamol is not recommended alone but is recommended in association with weak opioids for LBP (National Institute for Health and Clinical Excellence, 2016) (Table 1). For OA, both the American College of Rheumatology (ACR) and NICE recommend paracetamol to patients if NSAIDs and/or other pharmacological treatments fail (Kolasinski et al., 2019; National Institute for Health and Care Excellence, 2022a). Osteoarthritis Research Society International (OARSI), by contrast, does not recommend paracetamol in OA, given little to no efficacy with possible hepatotoxicity (Bannuru et al., 2019). Two geriatric societies—the American Geriatric Society (AGS) (American Geriatrics Society Panel on Pharmacological Management of Persistent Pain in Older Persons, 2009) and British Geriatric Society (BGS) (Abdulla et al., 2013)—recommend paracetamol for musculoskeletal pain in general among older adults (i.e., over 65 years). The two guidelines however have not been updated recently as they were released in 2009 and 2013 respectively.

**TABLE 1:** Guidelines on the use of analgesics for RMDs published or updated in the past 5 years.

Condition	Organisation/society	First author, latest updated year	Recommendation	Comments
LBP	NICE	National Institute for Health and Clinical Excellence (2016), 2020	Paracetamol (CR)	Not recommended alone, recommended in association with weak opioids
			NSAIDs (R)	
			Weak opioids (CR)	Recommended for acute LBP if NSAIDs fails
				Not recommended routinely for acute LBP
				Not recommended for chronic LBP
Acute LBP	American College of Physicians	Chou et al. (2017)^a^, 2017	NSAIDs	Small effects
Chronic LBP			NSAIDs	Small to moderate effects
			Opioids	Tramadol with modest effects; others with small effects
			Duloxetine	Small effects
AxSpA	ASAS-EULAR	van der Heijde et al. (2016), 2016	Paracetamol (CR)	To be considered after NSAIDs failed
			NSAIDs (R)	
			Opioids (CR)	To be considered after NSAIDs failed
OA	ACR	Kolasinski et al. (2019), 2020	Paracetamol (CR)	Recommended for patients intolerant to NSAIDs, monitor liver function
			NSAIDs (R)	
			Tramadol (CR)	Recommended for patients intolerant to NSAIDs
			Duloxetine (CR)	Recommended for patients intolerant to NSAIDs
OA	NICE	National Institute for Health and Care Excellence, (2022b), 2022	Paracetamol (CR)	To be considered after other pharmacological treatments failed
				Only infrequent use for short-term pain relief
			NSAIDs (R)	
			Weak opioids (CR)	To be considered after other pharmacological treatments failed
				Only infrequent use for short-term pain relief
OA	OARSI	Bannuru et al. (2019), 2019	Paracetamol (CNR)	Given a little to no efficacy in OA, with a signal for possible hepatotoxicity
			NSAIDs (R)	Not recommended (oral) for patients with cardiovascular comorbidities or frailty
			Duloxetine (CR)	To be considered for OA patients with widespread pain or depression
RA	NICE	National Institute for Health and Care Excellence, (2018), 2020	NSAIDs (R)	To be considered carefully for patients taking low-dose aspirin
Early arthritis	EULAR	Combe et al. (2016), 2017	NSAIDs (R)	To evaluate gastrointestinal, renal and cardiovascular risks before initiation
Chronic primary pain (e.g., fibromyalgia)	NICE	National Institute for Health and Care Excellence (2021a), 2021	Antidepressants (R)	Antidepressants include amitriptyline, citalopram, duloxetine, fluoxetine, paroxetine or sertraline
				To seek specialist advice if prescribing for young people aged 16–17 years

^a^ This guideline reports effectiveness rather than recommendations.

Abbreviations: ACR: american college of rheumatology; AxSpA: axial spondyloarthritis; CNR: conditionally not recommended; CR: conditionally recommended; EULAR: european alliance of associations for rheumatology; LBP: low back pain; NICE: national institute for health and care excellence; NSAIDs: Non-steroidal anti-inflammatory drugs; OA: osteoarthritis; OARSI: osteoarthritis research society international; R: recommended; RA: rheumatoid arthritis.

### Efficacy

There has been questionable effectiveness about the long-term use of paracetamol (Freo et al., 2021), with limited evidence supporting the efficacy of long-term use of paracetamol in RMDs (Abdel Shaheed et al., 2021). A systematic review showed paracetamol (4 g/day for 3–12 weeks) provided modest pain relief by 3.23 points on a 0–100-point pain scale (95% CI = −5.43, −1.02) for people with knee or hip OA (Leopoldino et al., 2019). The rest of the RMDs, by contrast, lack high-quality evidence on efficacy, in which chronic LBP, RA, non-cancer pain in children and adolescents, and neuropathic pain are supported by very low-quality evidence. More importantly, the evidence from a previous Cochrane review concluded that paracetamol (up to 4 g/day for up to 12 weeks) was not effective in reducing acute LBP (Saragiotto et al., 2016). In light of a short follow-up ranging from a few hours to 2 weeks after administration, most systematic reviews assessed the immediate treatment effect, making the effectiveness of paracetamol for chronic pain management in RMDs difficult to thoroughly evaluate.

### Safety

Whilst generally deemed fairly safe there has been emerging evidence of specific adverse effects in chronic use (McCrae et al., 2018). Regular long-term use at higher doses has been associated with an increased risk of gastrointestinal (GI) bleeding and a small increase in systolic blood pressure (BP) (2–4 mmHg) (Table 2). Regular use of daily doses of ≥2–3 g paracetamol was associated with a potentially increased risk of upper GI bleeding, with most being observational studies in participants aged 40 or older or in those with a history of ischemic stroke (García Rodríguez and Hernández-Díaz, 2001; González-Pérez and Rodríguez, 2006; Rahme et al., 2008; Doherty et al., 2011; Gonzalez-Valcarcel et al., 2016). An randomised controlled trial (RCT) also supported a decrease in haemoglobin (≥1 g/dl) at 13 weeks observed in 20.3% of participants on the treatment of paracetamol 3 g/day (Doherty et al., 2011). This effect was additive when combined with NSAIDs.

**TABLE 2:** Safety concerns about the use of analgesics.

First author, year	Article/study	Condition	Dosage/regimen	Comparator	Outcome	Relative effect (95% CI)	No of participants (studies)
Paracetamol—GI effects							
Gonzalez-Valcarcel et al. (2016)	Nested case-control	Patients with a history of ischemic stroke or transient ischemic attack	Oral; any use	Non-use	Major bleeding	OR 1.60 (1.26, 2.03)	809 cases vs. 1,616 controls
Doherty et al. (2011)	RCT	Community-derived people aged 40 + years with chronic knee pain	Oral; 13 weeks	—	Decrease in haemoglobin (≥1 g/dl)		892 (1)
			Paracetamol 3 g/day			20.3% (44/217)	
			Ibuprofen 1.2 g/day			19.6% (43/219)	
			Ibuprofen 600 mg/day + paracetamol 1.5 g/day			24.1% (53/220)	
			Ibuprofen 1.2 g/day + paracetamol 3 g/day			38.4% (83/216)—Twice than monotherapy (*p* < 0.001)	
Rahme et al. (2008)	Retrospective cohort	Age of 65 + years	Oral; >3 g/day	—^b^	GI hospitalisation rates	HR 1.20 (1.03, 1.40)	644,183 (1)
González-Pérez and Rodríguez, (2006)	Meta-analysis (case-control)	—	Oral; any use	Non-use	Upper GI complications	RR 1.3 (1.2, 1.5)	– (12)
García Rodríguez and Hernández-Díaz, (2001)	Nested case-control	Age of 40–79 years and without cancer, esophageal varices, Mallory-Weiss disease, liver disease, coagulopathies, and alcohol- related disorders	Oral; >2 g	Non-use	Upper GI complications	RR 3.6 (2.6, 5.1)	2,105 cases vs. 11,500 controls (1)
Paracetamol—BP effects							
Dawson et al. (2013)	Retrospective cohort	Patients with HTN aged 65 + years	Oral; any use	Non-use	Change in systolic BP (mmHg)	1.6 (0.7, 2.5)	2,754 acetaminophen-exposed
Sudano et al. (2010)	RCT	Coronary artery disease	3 g/day, 2 weeks	Placebo	Change in systolic BP (mmHg)	3	33
					Change in diastolic BP (mmHg)	2	
Forman et al. (2007)	Prospective cohort (2 years)	Male health professionals without HTN	6–7 days/week	Non-use	Incident HTN	RR 1.34 (1.00, 1.79)	16,031
Kurth et al. (2005)	Prospective cohort (14 years)	Men without HTN	Cumulative use over 14 years ≥2,500 pills	Non-use	Incident HTN	HR 1.08 (0.87, 1.34)	8,229
Dedier et al. (2002)	Prospective cohort (8 years)	Women aged 44–69 years without HTN or chronic renal insufficiency	1–4 days/month	Non-use	Incident HTN	OR 1.07 (1.02, 1.13)	51,630
			≥22			OR 1.20 (1.08, 1.33)	
Curhan et al. (2002)	Prospective cohort (2 years)	Women aged 31–50 years without HTN	1–4 days/month	Non-use	Incident HTN	RR 1.19 (1.04, 1.36)	80,020
			≥22			RR 2.00 (1.52, 2.62)	
						*p* for trend: <0.001	
Radack et al. (1987)	RCT	HTN	4 g/day, 3 weeks	Placebo	Change in systolic BP (mmHg)	0.2	15
Lewis et al. (1986)	Unblinded, three phase, crossover	HTN	4 g/day, 2 weeks	—	Change in systolic BP (mmHg)	−6.5 (mean arterial pressure)	21
		OA					
Chalmers et al. (1984)	RCT	HTN OA	3 g/day, 4 weeks	Placebo	Change in systolic BP (mmHg)	4	22
NSAIDs							
Bally et al. (2017)	Meta-analysis	Adults with acute myocardial infarction	Any dose for 1–7 days	Non-use	Acute myocardial infarction		446,763 (4)
			Celecoxib			OR 1.24 (0.91, 1.82)	
			Ibuprofen			OR 1.48 (1.00, 2.26)	
			Diclofenac			OR 1.50 (1.06, 2.04)	
			Naproxen			OR 1.53 (1.07, 2.33)	
			Rofecoxib			OR 1.58 (1.07, 2.17)	
Chan et al. (2017)	RCT (CONCERN)	Arthritis pain not relieved by basic analgesics Previous upper-GI bleeding during NSAID use Requirement for low-dose aspirin, or multiple CV risk factors	Celecoxib 100 mg BID (*n* = 257)	Naproxen 500 mg BID (*n* = 257)	Recurrent GI bleeding within 6 months	HR 0.44 (0.23, 0.82)	514
					Serious CV events at 6 months	HR 0.78 (0.36, 1.73)	
Nissen et al. (2016)	RCT (PRECISION)	Age 18 + years RA or OA requiring daily NSAIDs with high CV risk/established CV disease	Celecoxib 100 mg BID (*n* = 8,072)	Naproxen 375 mg BID (*n* = 7,969) or ibuprofen 600 mg TID (*n* = 8,040)	First occurrence of APTC event composite (non-inferiority)	celecoxib vs. naproxen: HR 0.93 (0.76, 1.12)	24,081
						celecoxib vs. ibuprofen HR 0.85 (0.70, 1.04)	
						ibuprofen vs. naproxen HR 1.08 (0.90, 1.31)	
					Clinically significant GI event	celecoxib vs. naproxen HR 0.97 (0.67, 1.40)	
						celecoxib vs. ibuprofen HR 0.76 (0.53, 1.08)	
					Clinically significant GI event + iron-deficiency anemia of GI origin event	celecoxib vs. naproxen HR 0.71 (0.54, 0.93)	
						celecoxib vs. ibuprofen HR 0.65 (0.50, 0.85)	
Combe et al. (2009)	RCT (MEDAL)	Age 50 + yearsRA or OA requiring chronic NSAIDs	Etoricoxib 90 mg once daily (*n* = 11,787)	Diclofenac 75 mg BID (*n* = 11,717)	Thrombotic CV-event composite (non-inferiority)	HR 0.96 (0.81, 1.15)	23,504
					Discontinuations due to GI adverse events	HR 0.84 (0.63, 1.13)	
Opioids—CNCP							
Nury et al. (2022)	Meta-analysis (most RCTs)	CNCP and chronic LBP	Opioids from 4 to 15 weeks	Placebo	Any adverse events	RR 1.20 (1.13, 1.28)	— (13) (low^a^)
					Nausea	RR 1.86 (1.35, 2.56)	— (13) (very low^a^)
					Vomiting	RR 3.26 (2.08, 5.09)	— (11) (low^a^)
					Constipation	RR 2.73 (1.98, 3.77)	— (13) (low^a^)
					Dizziness	RR 2.91 (2.17, 3.90)	— (10) (low^a^)
					Somnolence	RR 3.47 (2.33, 5.17)	— (10) (low^a^)
Busse et al. (2018)	Meta-analysis (RCTs)	CNCP	Opioids from 1.5 to 4 months	Placebo	Incidence of vomiting	RR 2.50 (1.89, 3.30)	5,961 (18)
Megale et al. (2018)	Meta-analysis (RCTs)	Older adults with musculoskeletal pain	Opioids	Placebo	Adverse events	OR 2.94 (2.33, 3.72)	–(23)
					Treatment discontinuation due to adverse events	OR 4.04 (3.10, 5.25)	
Opioids—RA							
Ozen et al. (2019)	Prospective	RA aged 40 + years without prior fracture	Weak opioids	Non-use	Incident fractures (vertebra, hip, forearm and humerus)	HR 1.37 (1.18, 1.59)	11,412 (1)
			Strong opioids			HR 1.53 (1.24, 1.88)	
Anastasiou et al. (2019)	—^b^	SLE and RA	—	—	Admissions due to opioid overdose	SLE: RR 2.44 (1.99, 2.98)	Of 33,207,455 hospitalizations, 512,740 (1.5%) with RA and 147,480 (0.44%) with SLE
						RA: RR 1.47 (1.30, 1.67)	
						Ref = neither condition	
Whittle et al. (2013)	Meta-analysis (RCTs)	RA	Opioids up to 6 weeks	Placebo	Avoid harm (No. of withdrawals due to adverse events)	RR 0.86 (0.79, 0.93) (favours placebo)	324 (3)
Whittle et al. (2011)	Meta-analysis (RCTs or CCTs)	RA	Opioids up to 6 weeks	Placebo	Withdrawal due to adverse events	RR 2.67 (0.52, 13.75)	331 (3) (low^a^)
					Report adverse events	OR 3.90 (2.31, 6.56)	371 (4) (low^a^)
Opioids—OA							
Kawai et al. (2022)	RCT	OA	Tramadol 100–300 mg/d, 4 weeks	Placebo	Any adverse events	Tramadol: 38.5% (30/78)	159 (1)
						Placebo: 13.6% (11/81)	
Krebs et al. (2018)	RCT	OA	Opioids, 12 months	Non-opioids	Adverse medication-related symptoms	Opioids: mean (SD) = 1.8 (2.6)	240 (1)
						Non-opioids: mean (SD) = 0.9 (1.8)	
						Between-Group Difference = 0.9 (95% CI = 0.3, 1.5) (overall *p* = 0.03)	
Serrie et al. (2017)	RCT	OA	Tapentadol PR 50–250 mg BID, oxycodone CR 10–50 mg BID; 15 weeks	Placebo	Any adverse event	Placebo: 55.5% (187/337)	990 (1)
						Tapentadol: 67.1% (214/319)	
						Oxycodone: 84.9% (281/331)	
					Any adverse event causing study discontinuation	Placebo: 8% (27/337)	
						Tapentadol: 18.8% (60/319)	
						Oxycodone: 42.3% (140/331)	
Etropolski et al. (2014)	Meta-analysis (RCTs)	OA	Tapentadol ER 100–250 mg BID, oxycodone CR 20–50 mg BID; from 15 weeks to 1 year	Placebo	Incidences of GI treatment-emergent adverse event	Placebo: 26.6% (264/993)	4,091 (1)
						Tapentadol: 47.3% (887/1874)	
						Oxycodone: 65.4% (800/1224)	
					Incidences of nervous system treatment-emergent adverse event	Placebo: 22.5% (223/993)	
						Tapentadol: 42.6% (799/1874)	
						Oxycodone: 45.1% (552/1224)	
Antidepressants							
Ferraro et al. (2021)	Meta-analysis (RCTs)	Adults with LBP	Any use	Placebo	Acceptability (all-cause discontinuation)	OR 1.27 (1.03, 1.56)	– (14)
					Tolerability (discontinuation due to adverse effects)	OR 2.39 (1.71, 3.34)	– (10)
Ferreira et al. (2021)	Meta-analysis (RCTs)	Participants with LBP, neck pain, sciatica, or hip or knee OA	SNRI	Placebo	Any adverse event^c^	RR 1.23 (1.16, 1.30)	– (13) (low^a^)
			SSRI			RR 1.53 (0.19, 12.61)	– (2) (very low^a^)
			TCAs			RR 1.49 (0.95, 2.34)	– (8) (low^a^)
			Tetracyclic antidepressants			RR 0.96 (0.79, 1.16)	– (1) (low^a^)
			NDRI			RR 2.80 (1.30, 6.02)	– (1) (low^a^)
Hauser et al. (2012)	Meta-analysis (RCTs)	Patients with fibromyalgia syndrome	SNRI	Placebo	Acceptability (total treatment discontinuation rates)	RR 0.98 (0.84, 1.14)	6,063 (10)
			SSRI			RR 1.36 (0.79, 2.36)	414 (7)
			TCAs			RR 0.76 (0.54, 1.07)	708 (11)
			SNRI	Placebo	Tolerability (dropout rates due to adverse events)	RR 1.83 (1.53, 2.18)	6,509 (10)
			SSRI			RR 1.60 (0.84, 3.04)	392 (7)
			TCAs			RR 0.84 (0.46, 1.52)	691 (10)
Gabapentinoids							
Torrance et al. (2020)	Trend	Individuals with at least one prescription for gabapentin or pregabalin	Gabapentinoids	Non-use	Age-standardised death rate	RR 2.16 (2.08, 2.25)	785,800 (1)

^a^ Certainty of evidence.

^b^ Information was not available due to a lack of full-text, or just a published abstract.

^c^ Adverse events were defined by each study and varied noticeably across trials, such as nausea (most prevalent), somnolence, back pain, diarrhoea, dizziness, dyspnea, muscular weakness, non-cardiac chest pain, hypoaesthesia, transient ischaemic attack, myocardial infarction, hypertensive encephalopathy, osteoarthritis and so on.

Abbreviations: APTC: Antiplatelet Trialists’ Collaboration; BID: two times a day; BP: blood pressure; CCT: Quasi-randomized controlled trial; CI: confidence interval; CNCP: Chronic non-cancer pain; CR: controlled release; ER: extended release; GI: gastrointestinal; HR: hazard ratio; HTN: hypertension; MACE: major cardiovascular events; MD: mean difference; NDRI: Noradrenaline-dopamine reuptake inhibitors; OR: odds ratio; PR: prolonged release; RCT: randomised controlled trial; RR: risk ratio; SLE: systemic lupus erythematosus; SNRI: Serotonin-noradrenaline reuptake inhibitors; SSRI: selective serotonin reuptake inhibitors; TCA: tricyclic antidepressants; TID: three times a day; WOMAC: Western Ontario and McMaster Universities Osteoarthritis Index.

BP increase has been another emerging concern with paracetamol (Turtle et al., 2013). The earliest study published in 1984 found an average of 4 mmHg increase in systolic BP when 3 g of paracetamol daily was administered for 4 weeks among patients with hypertension or OA (Chalmers et al., 1984). Subsequent RCTs had small sample sizes and showed inconsistent results, of which some supported an increase in BP (Radack et al., 1987; Sudano et al., 2010) but some against (Lewis et al., 1986). Similarly, most of the observational studies suggested that paracetamol in long-term use increased the risk of developing hypertension (Curhan et al., 2002; Dedier et al., 2002; Forman et al., 2007), with some conflicting evidence (Kurth et al., 2005; Dawson et al., 2013). Whilst several observational studies showed an association, the pain was often not measured and adjusted accordingly. An important confounding—uncontrolled pain could lead to high BP—of observational studies, however, would underestimate the association between paracetamol use and the change in BP, given the baseline BP might be higher. This could possibly explain the non-significant finding of the change in systolic BP in a cohort study (Dawson et al., 2013). The most recent NICE guideline defines hypertension as 140/90 mmHg and above, with a 10% or greater risk of developing cardiovascular disease (CVD) within the next 10 years (The Lancet, 2019; National Institute for Health and Care Excellence, 2019). A small increase in BP may be clinically important, especially for those with an increased baseline CVD risk. However, studies to date do not demonstrate these modest increase in BP has led to an increase in clinical endpoints such as stroke or myocardial infarctions (Table 2).

## Non-steroidal anti-inflammatory drugs (NSAIDs)

NSAIDs have been approved to be prescribed for a variety of conditions, including OA, RA, AxSpA, migraine, and mild to moderate acute/chronic pain, within different guidelines (Combe et al., 2016; National Institute for Health and Clinical Excellence, 2016; van der Heijde et al., 2016; National Institute for Health and Care Excellence, 2018; Bannuru et al., 2019; Kolasinski et al., 2019; Mei et al., 2020; National Institute for Health and Care Excellence, 2022b). The efficacy of NSAID treatments is indisputable, this review therefore will not put too much emphasis on it. Chronic NSAID use is defined as taking NSAIDs more than three times a week for more than 3 months. NSAIDs in chronic use have notably been reported for more than 29 million American adults (Zhou et al., 2014). The selection of an appropriate NSAID depends on patients’ profile, potential adverse effects, pharmacokinetic/pharmacodynamic properties, cost, and availability. The long-term use of NSAIDs has been associated with CV, GI, renal, skeletal muscle (e.g., interfere with muscle repair and fracture healing) and liver risks (Marcum and Hanlon, 2010; Sostres and Lanas, 2016; Mei et al., 2020). In recent years, there has been new evidence on the safety between COX2-selective and non-selective NSAIDs for chronic pain management, with a focus on GI and CV risks (Ho et al., 2018), which will be discussed in the next section.

### Safety

The PRECISION study (Nissen et al., 2016) was the first study using NSAIDs in high-CV-risk patients with OA or RA to assess the CV risk of COX2-selective (i.e., celecoxib 200 mg/day) and non-selective NSAIDs (i.e., ibuprofen 1,800 mg/day or naproxen 750 mg/day) (Table 2). Similar CV-event rates were observed between celecoxib vs. naproxen and ibuprofen (hazard ratio (HR) for celecoxib vs. naproxen: 0.90, 95% CI = 0.71, 1.15; HR for celecoxib vs. ibuprofen: 0.81, 95% CI = 0.65, 1.02), but GI tolerability was better for celecoxib (serious GI events: HR for celecoxib vs. naproxen: 0.71, 95% CI = 0.54, 0.93; HR for celecoxib vs. ibuprofen: 0.65, 95% CI = 0.50, 0.85) (Nissen et al., 2016). The risk of renal events was significantly lower with celecoxib than with ibuprofen but not with naproxen. The MEDAL program (Combe et al., 2009) that evaluated long-term use of COX2-selective NSAIDs also reported no difference in risk of thrombotic CV events in arthritis patients on long-term therapy with etoricoxib (90 mg/day) compared to diclofenac (150 mg/day). Despite the reassuring results, an increase in BP was noticed in both groups, and the rate of discontinuation due to hypertension was higher in the etoricoxib group (Combe et al., 2009). The CONCERN study (Chan et al., 2017) assessed the risk of GI events between COX2-selective (i.e., celecoxib 200 mg/day) and non-selective NSAIDs (i.e., naproxen 1,000 mg/day), both in combination with a prophylactic proton pump inhibitor. This trial included arthritis patients who had cardiothrombotic diseases requiring low-dose aspirin and a history of upper-GI-tract bleeding and followed up recurrent upper-GI-tract bleeding for 18 months. Celecoxib was found to be associated with fewer adverse GI-tract events than naproxen (HR = 0.44, 95% CI = 0.23, 0.82).

Over the last two decades, an increase in CVD risk has been the major concern with NSAIDs especially following two COX2-selective NSAIDs, rofecoxib and valdecoxib, were withdrawn from the market in 2004 and 2005 and deemed unsafe (U.S. Food and Drug Administration, 2018). Additionally in 2013, following a Europe-wide review of CVD safety, diclofenac was issued safety warnings and contraindicated in patients with established ischaemic heart disease, peripheral arterial disease, cerebrovascular disease and congestive heart failure (Medicines and Healthcare products Regulatory Agency, 2014; Coxib and traditional NSAID Trialists' (CNT) Collaboration Emberson et al., 2013; Schmidt et al., 2018). Regarding the newer COX2-selective NSAIDs, reassuringly a meta-analysis (Bally et al., 2017) showed no significant difference in the rate of acute myocardial infarction between celecoxib and non-selective NSAIDs, and celecoxib was the only COX2-selective NSAID with a lower risk of adverse CV and GI events compared with non-selective NSAIDs. The risk was greatest during the first month of NSAID use and with higher doses. Summarising the latest evidence it indicates that long-term use of celecoxib 200 mg/day may be considered for patients at increased CV risk, given the comparable risk of CV events and favourable profile of GI adverse events compared to non-selective NSAIDs (Bally et al., 2017). The 2007 scientific statement from the American Heart Association, however, advised that COX2 inhibitors should be used at the lowest possible dose and for the shortest possible time to minimise the risk of CV events until more long-term data on CV safety is available (Antman et al., 2007). In patients where vascular risks are a concern, the safest option appears to be naproxen when compared to other NSAIDs (Coxib and traditional NSAID Trialists' (CNT) Collaboration Emberson et al., 2013), emphasising the need to personalise the approach to prescribing all analgesics based on an individual’s baseline risk.

## Opioids

Opioid prescribing is increasing for chronic non-cancer pain (CNCP) in high-income countries over the last few decades (Jani et al., 2020; Jani et al., 2021). Whilst use in acute pain has been well established, chronic/long-term use has been subject to considerable controversy in recent years due to its downstream adverse outcomes (Kalso et al., 2004).

### Efficacy

Recent evidence from a meta-analysis suggests that opioid use was associated with statistically significant but small clinical improvements in pain and physical functioning/disability among people with CNCP, accompanied by a higher risk of adverse effects (Busse et al., 2018). Opioid use was associated with reduced pain [weighted mean difference (WMD) = −0.69 cm (95% CI = −0.82, −0.56) on a 10-cm visual analogue scale] and improved physical functioning [WMD = 2.04 points (95% CI = 1.41, 2.68) on the 100-point 36-item short form physical component score] compared to the placebo (Busse et al., 2018). Compared with non-opioid alternatives including NSAIDs, tricyclic antidepressants (TCAs), or anticonvulsants, opioids showed similar associations with improvements in pain and physical functioning, with low-to moderate-quality evidence only (Busse et al., 2018). Another meta-analysis focusing on chronic LBP reported the short-term (4–15 weeks) use of strong opioids might have clinically relevant reductions in pain compared to placebo (Nury et al., 2022). However even short-term use was associated with increases in GI and nervous system adverse events.

Despite frequent long-term opioid use for CNCP management in RMDs, there has been no scientific evidence to support its efficacy but with increasing evidence of adverse events in this population (Anastasiou and Yazdany, 2022). To date, there is limited evidence on the efficacy and safety of opioid use in RA and SLE, with scarce data in other RMDs (Anastasiou and Yazdany, 2022). Research on RA cohorts supported weak opioids in short-term (<6 weeks) use for pain control, with a relative risk (RR) = 1.40 (95% CI = 1.07, 1.85) that favours opioids over placebo (Whittle et al., 2013). Opioids were also superior to placebo in RA patient-reported global impression of change, with an absolute risk difference of 18% (95% CI = 1, 41), a relative percent change of 44% (95% CI = 3, 103), and numbers needed to treat (NNT) as 6 (95% CI = 3, 84) (Whittle et al., 2011). In OA, an RCT examining chronic opioid therapy for moderate to severe chronic back pain or hip or knee OA found no significant difference in pain-related function over 12 months (Krebs et al., 2018). The pain intensity was however significantly better in the non-opioid analgesic group which also had fewer adverse medication-related symptoms (Krebs et al., 2018). For inflammatory RMDs that require biologic disease-modifying antirheumatic drugs (DMARDs) treatments, early opioids may improve pain in the short term, resulting in delayed DMARD initiation or reduced DMARD use (Boytsov et al., 2019; Kimsey et al., 2019).

### Safety

Opioid use was related to increased vomiting (RR = 2.50, 95% CI = 1.89, 3.30) among people with CNCP (Busse et al., 2018). Opioid treatments also showed 3 times higher odds of adverse events (OR = 2.94, 95% CI = 2.33, 3.72) and 4 times higher odds of treatment discontinuation due to adverse events (OR = 4.04, 95%CI = 3.10, 5.25) among older adults with musculoskeletal pain (Megale et al., 2018).

There has been limited RMD-specific research on opioid safety for pain management, in which RA and OA are the most commonly studied. For RA patients, opioids in short-term use were more likely to report adverse events such as nausea, vomiting, dizziness and constipation (OR = 3.90, 95% CI = 2.31, 6.56) (Whittle et al., 2011), but the risk of withdrawals due to adverse events was inconsistent between studies (Whittle et al., 2011; Whittle et al., 2013). Opioid use was also associated with an increased risk of fracture in the RA cohort, with a greater risk observed in strong opiates (HR = 1.53, 95% CI = 1.24, 1.88) than in weak opiates (HR = 1.37, 95% CI = 1.18, 1.59) (Ozen et al., 2019). Moreover, both RA and systemic lupus erythematosus (SLE) patients had a higher risk of hospital admissions due to opioid overdose compared to other hospitalisations (Anastasiou et al., 2019). Regarding OA, several RCTs have shown that patients receiving opioid treatments experience more adverse events and have higher proportions of dropout due to adverse events than those on placebo or non-opioids (Etropolski et al., 2014; Serrie et al., 2017; Krebs et al., 2018; Kawai et al., 2022). In the 12th month, patients on opioids had a significant increase in medication-related symptoms by 0.9 (95% CI = 0.3, 1.5) compared to those on non-opioid treatments (Krebs et al., 2018). Previous work also suggested that tapentadol PR seemingly had a better GI tolerability profile (GI adverse event = 47.3%) than oxycodone CR (GI adverse event = 65.4%) (Etropolski et al., 2014; Serrie et al., 2017) (Table 2).

To date, there are only a few studies investigating long-term opioid therapy for more than 6 months, with no study following up more than 1 year (Chou et al., 2015; Krebs et al., 2018; Nury et al., 2022). Long-term opioid therapy (defined in this study as ≥6 months) for CNCP appears not to be superior to non-opioids in improvements of pain, disability, or pain-related function but shows more adverse events, including treatment discontinuation, opioid abuse or dependence and all-cause mortality (Krebs et al., 2018; Nury et al., 2022). In addition, there is no study evaluating the long-term effectiveness of different opioid dosing strategies such as short- plus long-acting opioids vs. long-acting opioids alone (Chou et al., 2015). In light of a lack of evidence demonstrating consistently improved pain control with long-term opioid use in RMDs but with increased risks of adverse events, the current evidence strongly suggests that opioids do not have a routine role in the CNCP management of inflammatory rheumatic diseases (Anastasiou and Yazdany, 2022).

## Antidepressants

### Chronic pain conditions (except fibromyalgia)—Efficacy and safety

Antidepressants for the treatment of LBP (Ferraro et al., 2021) and OA (Ferreira et al., 2021) have been found associated with small reductions in pain intensity or disability scores but the effect on pain might not be clinically important. In the meta-analysis assessing LBP (Ferraro et al., 2021), antidepressants showed a reduction of 4.33 points (95% CI = −6.15, −2.50, on a 0–100 scale) in pain intensity but had increased odds of stopping treatment for any reason (OR = 1.27, 95% CI = 1.03, 1.56) or due to adverse effects (OR = 2.39, 95% CI = 1.71, 3.34). In the meta-analysis evaluating back pain and OA (Ferreira et al., 2021), serotonin and norepinephrine reuptake inhibitors (SNRIs) reduced back pain by 5.30 points (95% CI = −7.31, −3.30, on a 0–100 scale, moderate evidence level) and OA pain by 9.72 points (95% CI = −12.75, −6.69, low evidence level) at 3–13 weeks. SNRIs were also found to decrease disability from back pain at 3–13 weeks (−3.55, 95% CI = −5.22, −1.88) and disability due to OA at 2 weeks or less (−5.10, 95% CI = −7.31, −2.89), with moderate evidence level respectively (Ferreira et al., 2021). Despite the efficacy, SNRIs were related to a high risk of adverse effects (RR = 1.23, 95% CI = 1.16, 1.30). Adverse events in this meta-analysis referred to various symptoms such as nausea (most prevalent), somnolence, back pain, diarrhoea, dizziness, transient ischaemic attack, and myocardial infarction, because they were defined by each study and varied noticeably across trials (Skljarevski et al., 1976; Skljarevski et al., 2009; Ferreira et al., 2021). TCAs and other antidepressants, by contrast, did not reduce pain or disability from back pain and had no available information about the treatment of OA.

The current evidence on the efficacy of antidepressants for musculoskeletal pain appears conflicting, leading to the discrepancy in guideline recommendations. NICE does not recommend the use of antidepressants for LBP (National Institute for Health and Clinical Excellence, 2016), while the American College of Physicians guidance suggests considering duloxetine as a second-line drug treatment for chronic LBP (Chou et al., 2017). Similarly, NICE does not make a specific recommendation on antidepressants for OA (National Institute for Health and Care Excellence, 2022b) but OARSI guidance makes a conditional recommendation on duloxetine for people with OA and widespread pain or depression (Bannuru et al., 2019). Despite the small effects at the group level reported in the present evidence, some treated individuals may gain a worthwhile benefit from antidepressants. For example, absolute effect sizes for physical treatments for LBP are of similar magnitudes to those reported in the previous review (Ferreira et al., 2021) and translate into NNT of between 5 and 9 (Froud et al., 2009; Underwood and Tysall, 2021).

### Fibromyalgia—Efficacy and safety

Antidepressants such as amitriptyline, citalopram, duloxetine, fluoxetine, paroxetine or sertraline are suggested by NICE guidelines to manage chronic primary pain (e.g., fibromyalgia) for adults (National Institute for Health and Care Excellence, 2021a). Most antidepressants are off-label use since, to date, only three pharmacological agents—pregabalin (i.e., gabapentinoids), duloxetine and milnacipran—approved by the United States Food and Drug Administration to treat fibromyalgia. A meta-analysis evaluated the efficacy and harms of antidepressants in the management of fibromyalgia syndromes (Hauser et al., 2012). The standardised mean differences of SNRIs, selective serotonin reuptake inhibitors (SSRIs), and TCAs on pain, sleep, fatigue, depression and health-related quality of life were all significant, despite only a small effect size reported. The NNT was estimated as 10.0 (95% CI = 8.0, 13.4) for SNRIs, 6.3 (95% CI = 4.1, 14.1) for SSRIs, and 4.9 (95% CI = 3.5, 8.0) for TCAs (Hauser et al., 2012). The RR of dropouts due to adverse events was higher for SNRIs (1.83, 95% CI = 1.53, 2.18) but was not statistically different for SSRIs and TCAs (Hauser et al., 2012). Although antidepressants are increasingly prescribed for fibromyalgia as per guideline recommendations, physicians and patients should be realistic about the balance between the benefits and harms of antidepressants.

The inconsistent recommendations of antidepressants across different guidelines are challenging, given the limited evidence on antidepressants’ efficacy for different RMDs. The current evidence indicates that for non-fibromyalgia RMDs, antidepressants have no important benefit that is less acceptable, less safe, and less tolerable (Ferraro et al., 2021).

## Gabapentinoids

There is well-established evidence supporting the use of gabapentin in people with postherpetic neuralgia and peripheral diabetic neuropathy for pain relief (Moore et al., 2014; Wiffen et al., 2017). Around 3-4 out of 10 participants achieved at least 50% pain intensity reduction with gabapentin, compared with 1-2 out of 10 for placebo (Wiffen et al., 2017). Evidence for chronic non-neuropathic pain conditions, however, is very limited, making it difficult to discuss the efficacy and safety of gabapentinoids thoroughly in this review. The previous RCT showed that gabapentin was not superior to placebo in the reduction in pain intensity and disability scores for chronic LBP (Atkinson et al., 2016). Despite the unclear efficacy of gabapentinoids in most pain conditions, its’ prescribing rate has increased drastically between 2006–2016 in Scotland, with a 4-fold increase for gabapentin and 16-fold for pregabalin (Torrance et al., 2020). The increasing prescribing is in line with the findings of gabapentinoids being increasingly abused or misused to self-medicate, in which opioid use disorder is the greatest risk factor (Evoy et al., 2021). Emerging evidence therefore reports the harms of gabapentinoids in terms of hospital utilisation and mortality risk (Evoy et al., 2021). People prescribed gabapentinoids had doubled the age-standardised death rate than that in the Scottish population (RR = 2.16, 95% CI = 2.08, 2.25) (Torrance et al., 2020). The increase in gabapentinoid prescribing, along with frequent co-prescriptions of opioids and/or benzodiazepines, also contributed to a higher rate of drug-related deaths (Torrance et al., 2020).

Gabapentinoids appear not to be recommended for RMDs by various guidelines given their unclear efficacy but do reveal an increasing prescribing rate and associations with poor health outcomes. The early evidence indicates some concerns regarding the appropriateness of gabapentinoids for chronic non-neuropathic pain management.

## Conclusion

This narrative review incorporates heterogeneous study designs for RMDs with detailed interpretation and discussion, aiming to provide up-to-date and well-evidenced-based information to healthcare professionals on a topic with little evidence. Limitations of this work should also be acknowledged: 1) data unavailability due to no full-text, or a format of published abstract 2) incomparability of studies because of heterogeneous dose regimens, comparators, or outcomes.

In this review, we have discussed some of the challenges in interpreting the evidence and following current guidelines, which inevitably lead to variation in clinical practice when prescribing analgesics to patients with RMDs. The heterogeneity in the quality of clinical trials to assess the analgesic efficacy and a lack of key metrics such as NNT and number needed to harm, make the interpretability of evidence difficult. This subsequently makes communications with patients about the benefit/harm balance and shared decision-making more challenging. Whilst population-level estimates can help identify subgroups of patients at high risk of specific adverse outcomes, prescribing often requires a personalised approach that incorporates the baseline risk of the patient as well as patient preference. Long-term use of opioids, antidepressants and gabapentinoids prescribed frequently in RMDs is importantly associated with dependence, often with minimal clinical benefits in symptoms or function. Recognising these challenges, NICE has released helpful resources to consider when prescribing dependence-forming medicines or antidepressants (National Institute for Health and Care Excellence, 2022a) as well as on shared decision-making with patients (National Institute for Health and Care Excellence, 2021b). Resources for effective non-pharmacological options for pain, alongside quantitative safety estimates that can be easily communicated with patients, would allow more informed choices and better treatment stratification than is currently possible.
